# Stability of commercial glucanase and *β*-glucosidase preparations under hydrolysis conditions

**DOI:** 10.7717/peerj.402

**Published:** 2014-06-10

**Authors:** Oscar Rosales-Calderon, Heather L. Trajano, Sheldon J.B. Duff

**Affiliations:** Department of Chemical and Biological Engineering, The University of British Columbia, Vancouver, BC, Canada

**Keywords:** Lignocellulosic ethanol, Enzymatic hydrolysis, β-glucosidase, Endoglucanase, Exoglucanase, Protein stability, Kinetic model

## Abstract

The cost of enzymes makes enzymatic hydrolysis one of the most expensive steps in the production of lignocellulosic ethanol. Diverse studies have used commercial enzyme cocktails assuming that change in total protein concentration during hydrolysis was solely due to adsorption of endo- and exoglucanases onto the substrate. Given the sensitivity of enzymes and proteins to media conditions this assumption was tested by evaluating and modeling the protein concentration of commercial cocktails at hydrolysis conditions. In the absence of solid substrate, the total protein concentration of a mixture of Celluclast 1.5 L and Novozyme 188 decreased by as much as 45% at 50 °C after 4 days. The individual cocktails as well as a mixture of both were stable at 20 °C. At 50 °C, the protein concentration of Celluclast 1.5 was relatively constant but Novozyme 188 decreased by as much as 77%. It was hypothesized that Novozyme 188 proteins suffer a structural change at 50 °C which leads to protein aggregation and precipitation. Lyophilized *β*-glucosidase (P-*β*-glucosidase) at 50 °C exhibited an aggregation rate which was successfully modeled using first order kinetics (*R*^2^ = 0.97). By incorporating the possible presence of chaperone proteins in Novozyme 188, the protein aggregation observed for this cocktail was successfully modeled (*R*^2^ = 0.96). To accurately model the increasing protein stability observed at high cocktail loadings, the model was modified to include the presence of additives in the cocktail (*R*^2^ = 0.98). By combining the measurement of total protein concentration with the proposed Novozyme 188 protein aggregation model, the endo- and exoglucanases concentration in the solid and liquid phases during hydrolysis can be more accurately determined. This methodology can be applied to various systems leading to optimization of enzyme loading by minimizing the excess of endo- and exoglucanases. In addition, the monitoring of endo- and exoglucanases concentrations can be used to build mass balances of enzyme recycling processes and to techno-economically evaluate the viability of enzyme recycling.

## Introduction

Low cost and readily available lignocellulosic materials, such as wheat straw, switchgrass, or poplar, are composed of cellulose and other polysaccharides that can be converted to ethanol. Biological processing of lignocellulose is attractive due to its mild operating conditions, and consists of four processing steps: (1) pretreatment of the raw material to expose the cellulose to enzymes, (2) enzymatic hydrolysis to convert polysaccharides to simple sugars, (3) fermentation of sugars to ethanol and (4) separation of ethanol from the fermentation broth (Bommarius et al., 2008).

Enzymatic hydrolysis is a complex reaction where exo- and endoglucanases or cellulases, most commonly from the fungus *Trichoderma reesei*, adsorb on cellulose to release glucooligomers, cellobiose, and glucose. As cellobiose inhibits cellulase activity, *β*-glucosidase is added to the hydrolysis to cleave cellobiose to glucose (Lu et al., 2002). However, as lignocellulose also contains non-hydrolysable lignin to which cellulases adsorb irreversibly, some cellulases are lost during hydrolysis (Farinas et al., 2010; Jørgensen, Kristensen & Felby, 2007; Zhou et al., 2009). These factors necessitate a high enzyme load, which increases hydrolysis costs; indeed one of the key economic factors and thus impediments to lignocellulosic ethanol production and commercialization is the amount and cost of enzyme needed for enzymatic hydrolysis (Lynd et al., 2008; Qi et al., 2011). Consequently, enzymatic hydrolysis has been the target of numerous studies (Jørgensen, Kristensen & Felby, 2007; Taherzadeh & Karimi, 2007). The hydrolysis is affected by multiple factors such as enzyme loading, reaction time, temperature, substrate composition, and inhibitor concentration. Several studies have attempted to increase the efficiency of the enzyme system or to minimize the amount of enzyme needed for hydrolysis (Arantes & Saddler, 2011; Shen & Agblevor, 2008). The recycling of cellulases bound to the residual substrate as well as enzymes in the reaction suspension is relatively new technology that has been gaining popularity because of its potential to decrease enzyme requirements during ethanol production (Qi et al., 2011).

In order to quantify the amount of enzymes that can be recycled, it is necessary to understand the concentration changes of endo- and exoglucanases, and *β*-glucosidase as well as proteins and compounds contained in the enzyme preparations used in the hydrolysis. Commercial enzyme cocktails from Novozyme: Celluclast 1.5 L and Novozyme 188 have been widely used as sources of endo- and exoglucanases, and *β*-glucosidase, respectively, for enzymatic hydrolysis (Arantes & Saddler, 2011; Hu et al., 2013; Kristensen et al., 2007; Qi et al., 2011; Rodrigues et al., 2012; Shi et al., 2011; Zheng et al., 2009) and enzyme recycling by adsorption research (Lu et al., 2002; Qi et al., 2011; Steele et al., 2005; Tu & Saddler, 2010; Tu, Chandra & Saddler, 2007a; Tu, Chandra & Saddler, 2007b). Endo- and exoglucanases present in solution after hydrolysis can be recycled by adsorption onto fresh substrate to start a new round of hydrolysis (Qi et al., 2011). Unfortunately, *β*-glucosidase cannot be simultaneously recycled by adsorption as it either does not adsorb to the substrate (Tu & Saddler, 2010; Tu, Chandra & Saddler, 2007a) or adsorbs to a far lesser degree than endo- and exoglucanases (Kumar & Wyman, 2009). Therefore fresh *β*-glucosidase must be added at the beginning of each round of hydrolysis to avoid build-up of cellobiose and subsequent end-product inhibition of cellulase (Qi et al., 2011). The newest commercial cellulase cocktails, such as Cellic CTec3 from Novozyme (Bagsværd, Denmark) and Accellerase TRIO from DuPont Genencor (CA, USA), contain a blend of endo- and exoglucanases and *β*-glucosidase. Therefore, the use of Celluclast 1.5 L and Novozyme 188 as enzyme sources are ideal for the enzyme recycling research, since *β*-glucosidase from Novozyme 188 can be used to complement recycled cellulases.

Many of the enzyme recycling and hydrolysis studies rely upon the assumption of constant protein concentration at hydrolysis conditions; however, this assumption has not been evaluated. Enzyme and protein structures determine solubility and activity and can change with pH, temperature, and/or concentration (Morris, Watzky & Finke, 2009). Therefore, the protein concentration in solution depends on system conditions and must be considered in enzyme adsorption and recycling studies. The objectives of this paper are to evaluate the stability of Celluclast 1.5 L and Novozyme 188 protein in solution and to model changes in protein concentration at enzymatic hydrolysis conditions in order to support the subsequent development of a mass balance and economic analysis for enzyme recycling.

## Materials & Methods

### Enzyme preparations

Lyophilized powder cellulases from *Trichoderma reesei* (P-cellulase, Cat. No. C8546, Sigma Aldrich) and *β*-glucosidase from *Aspergillus niger* (P-*β*-glucosidase, Cat. No. 9033-06-1, Sigma Aldrich) were used as reference enzymes. Commercial enzyme cocktails from Novozyme were also used: cellulase from *T. reesei*, Celluclast 1.5 L (129.3 mg protein/mL, 30.7 CBU/mL, 63.8 FPU/mL) and *β*-glucosidase derived from *A. niger*, Novozyme 188 (102.2 mg protein/mL, 626.4 CBU/mL). All enzymes were stored at 2 °C until use.

### Analysis of enzyme activity and protein concentration

Cellulase activity was measured following the NREL filter paper assay (Adney & Baker, 2008) and reported in filter-paper units (FPU) per milliliter of solution. *β*-glucosidase activity was measured using the method described by Wood & Bhat (1988) and reported in cellobiase units (CBU).

Protein concentration was measured by the Bio-Rad protein assay based on the colorimetric method of Bradford (Bio-rad, CA). This is a dye-binding assay in which the absorbance shift of the dye Coomassie Brilliant Blue G-250 is proportional to the protein concentration. As the Coomassie blue dye binds to primarily basic and aromatic amino acid residues, especially arginine, it is important to select the correct reference protein to accurately quantify protein (Zhu, Sathitsuksanoh & Zhang, 2009). Therefore, the potential of P-cellulase, P-*β*-glucosidase, and the widely used Bovine Serum Albumin (BSA) as calibration standards was studied.

Calibration standards with a final volume of 10 mL were prepared with Type II* water (Purelab) for each protein concentration at room temperature. Samples of 0.5 mL were taken and centrifuged (RCF 16,904 g, 10 min). The absorbance spectrum was measured for each sample from 200 to 700 nm (UV-1800, Shimadzu). The spectrum curves of the two enzymes were identical with three peaks at 264, 312 and 595 nm. Protein content was measured at 595 nm in the present study as it presented a higher and more defined response than those at 264 and 312 nm, and consequently increased measurement sensitivity.

**Figure 1:**
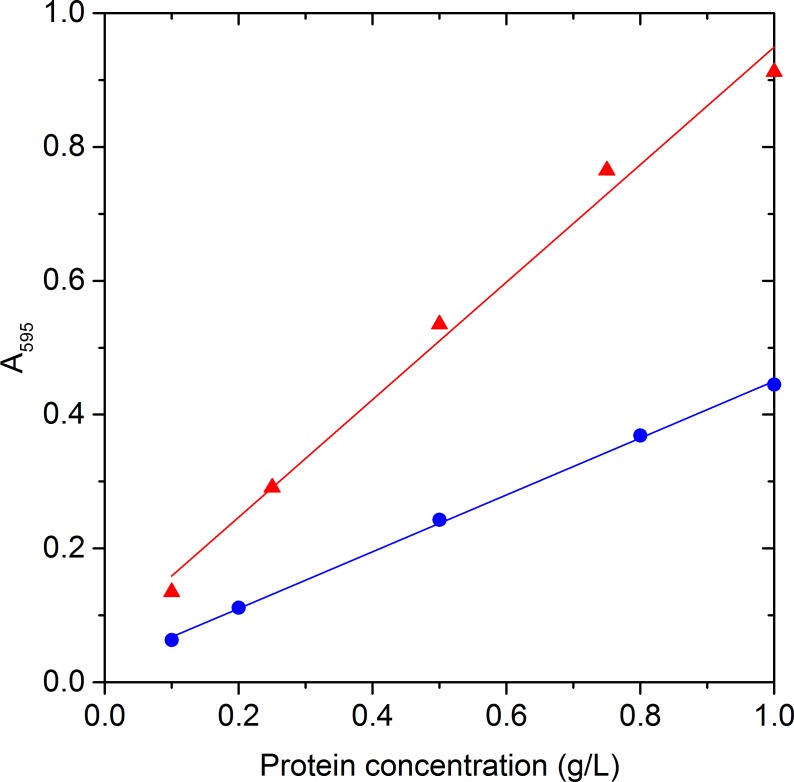
Protein calibration curves. Protein calibration curves (solid lines) for P-cellulase and P-*β*-glucosidase (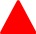); and Bovine Serum Albumin (BSA) (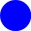).

The response of BSA was different from P-cellulase and P-*β*-glucosidase as shown in Fig. 1, due to differences in their amino acid compositions (Zhu, Sathitsuksanoh & Zhang, 2009). As the use of lyophilized enzymes as a calibration standard increases the accuracy in the protein quantification (Zhu, Sathitsuksanoh & Zhang, 2009), P-cellulase and P-*β*-glucosidase were used for this study instead of the widely used BSA.

### Pretreatment

Oxygen delignification pretreatment of wheat straw was conducted in a 1 L bench top reactor (PARR 4520). The reactor configuration allowed control of temperature, reaction time, NaOH concentration, and oxygen partial pressure. The reactor was charged at a concentration of 4% (w/w) dry wheat straw and 6–10% NaOH (w/w dry biomass), with a total mass of 500 g. The reactor was sealed and purged with nitrogen in order to remove oxygen. The vessel was heated to the desired temperature and then oxygen was bubbled through the reactor at 1 L/min. A mixing speed of 180 rpm was maintained during the entire process. The reaction was stopped by placing the reactor in ice and bringing it to atmospheric pressure. The pretreated biomass was then filtered and washed using a Buchner funnel and Whatman No. 4 filter paper. The substrates’ compositions are shown in Table 1.

**Table 1:** Composition of raw and pretreated substrates.

Pretreatment conditions	Composition (%)			Moisture content (%)	Substrate abbreviation
	Glucan	Xylan	Lignin		
Raw	35.8	20.6	15.8	6.9	R
30 min, 6% NaOH, 120 °C	49.9	23.6	9.0	81.7	M
60 min, 10% NaOH, 150 °C	55.3	24.2	4.7	82.0	S

### Protein stability

Protein stability was tested by quantifying protein concentration in solution over the course of 80 h. Acetate buffer (50 mM, pH 4.8) supplemented with 0.02% w/v tetracycline and 0.015% w/v cyclohexamide was placed in shakers (150 rpm) in 250 mL Erlenmeyer flasks at 20 or 50 °C for one hour in order to achieve thermal equilibrium before the desired amount of lyophilized enzymes or commercial enzyme preparation was added to a 50 mL final volume. Samples of 0.5 mL were withdrawn periodically and then centrifuged (RCF 16,904 g, 10 min). The protein concentration of the supernatant was measured.

Novozyme 188 protein stability in the presence of pretreated biomass was tested by quantifying the cocktail’s protein concentration in solution over the course of 120 h. Pretreated wheat straw (substrate S or M) was placed in the same buffer used in the initial tests at 5% wt solids concentration. The mixture was placed in a shaker (150 rpm) in 250 mL Erlenmeyer flasks at 50 °C before the desired amount of Novozyme 188 was added to a 50 mL final volume. Samples of 0.5 mL were withdrawn periodically and then centrifuged (RCF 16,904 g, 10 min) prior to determining protein content.

## Results and Discussion

### Stability of commercial enzyme preparations

**Figure 2:**
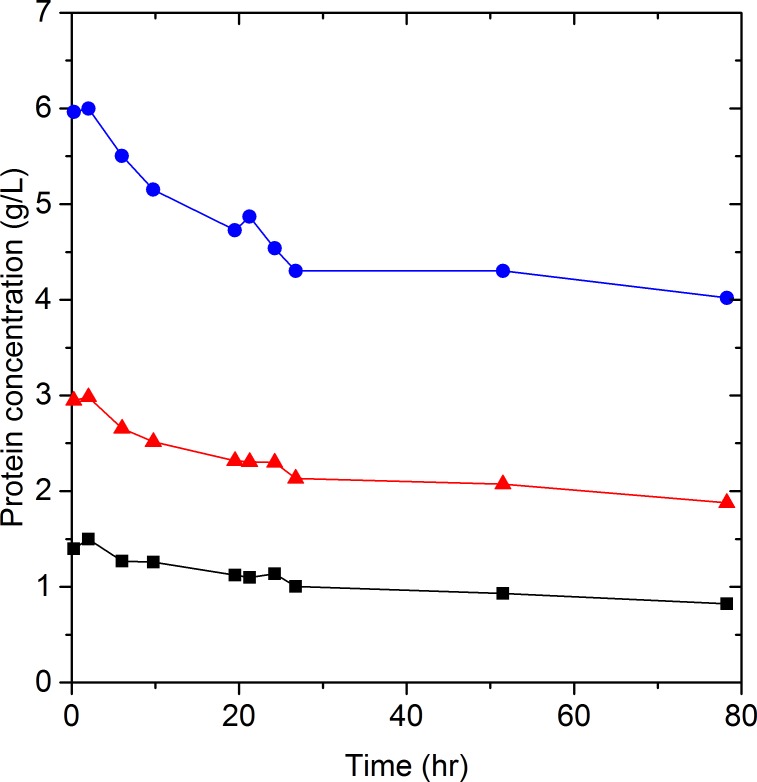
Celluclast 1.5 L and Novozyme 188 protein concentration as a function of time at 50 °C. Celluclast 1.5 L and Novozyme 188 protein concentration in the same solution at different initial concentrations [6 g/L (2.2 FPU/mL) 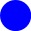, 3 g/L (1.1 FPU/mL) 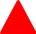 and 1.4 g/L (0.5 FPU/mL) 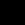] over time at 50 °C. Lines are added to assist in visualizing trends. The ratio of 5 CBU per FPU was consistent in all trials.

The enzyme system commonly used for enzymatic hydrolysis is a mixture of cellulases (endo- and exoglucanases) and *β*-glucosidases. Endo- and exoglucanases adsorb onto the substrate, primarily cellulose and lignin, while *β*-glucosidase has been reported to either not adsorb to the substrate (Tu & Saddler, 2010; Tu, Chandra & Saddler, 2007a), or to have a much lower adsorption on lignin than endo- and exoglucanases (Kumar & Wyman, 2009). Even though Celluclast 1.5 L and Novozyme 188 contain multiple proteins, enzymes and other compounds, numerous studies have assumed that any change in the total protein concentration during hydrolysis is due to the adsorption of endo- and exoglucanases on substrate (Arantes & Saddler, 2011; Lu et al., 2002; Qi et al., 2011; Tu, Pan & Saddler, 2009; Tu, Chandra & Saddler, 2007b). By this reasoning, the protein concentration of Celluclast 1.5 L and Novozyme 188 in the liquid phase should remain constant when substrate is absent. The validity of this assumption was tested by monitoring the protein concentration of Celluclast 1.5 L and Novozyme 188, for 78 h without substrate at 50 °C, a common hydrolysis temperature. As shown in Fig. 2, the protein concentration decreased by 30 to 45% dependent on the initial enzyme loading.

Farinas et al. (2010) reported the activity of endoglucanases and *β*-glucosidases from a wild-type strain of *A. niger*. All the enzymes showed high stability at 37 °C, however, the activity of *β*-glucosidases decreased by approximately 28, 20, and 45% after 48, 72 and 96 h incubation at 50 °C, respectively. In the same study, a 40 and 60% loss in activity after 24 and 96 h incubation at 50 °C was also reported for endoglucanases, which points to temperature as the responsible variable. In our work, precipitate was observed in the centrifuged samples prior to protein quantification and it appeared that the amount of precipitate increased with hydrolysis time. Given our observations and Farinas’s previous work, we hypothesized that high temperatures promote protein aggregation causing the concentration of protein in the liquid phase to decrease.

**Figure 3:**
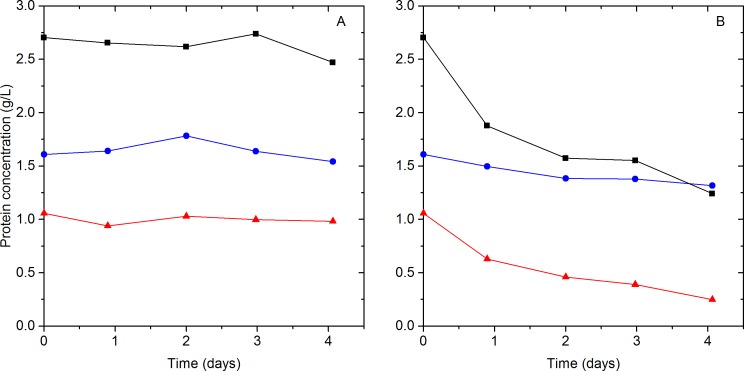
Cocktail protein concentration at 20 °C (A) and 50 °C (B). Celluclast 1.5 L and Novozyme 188 (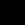), Celluclast 1.5 L (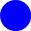) and Novozyme 188 (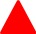) protein concentration as a function of time at 20 and 50 °C. Lines are added to assist in visualizing trends.

To confirm that aggregation is a consequence of temperature, and not other variables, such as shaking (Wang, 2005), Celluclast 1.5 L, Novozyme 188, and a mixture of the two were incubated statically at 20 °C and 50 °C. The protein concentration was monitored for four days as shown in Fig. 3. At 20 °C the protein concentration remained practically constant for Celluclast 1.5 L, Novozyme 188 and the mixture of the two cocktails (Fig. 3). However, at 50 °C a 34% drop in the total protein concentration was observed. Celluclast 1.5 L suffered an 18% decrease in its concentration at 50 °C after 4 days, demonstrating remarkable stability at high temperatures. The loss in Celluclast 1.5 L protein concentration is similar to the 14% precipitation reported by Chylenski et al. (2012) for a cellulase preparation from *T. reesei* after 124 h at 50 °C . In contrast, the protein concentration of Novozyme 188 decreased by 77% in 4 days at 50 °C. This decrease in concentration was accompanied by an increase in the quantity of solid matter on the bottom of the flasks, indicating protein precipitation. It is important to note that the arithmetic sum of the individual Celluclast 1.5 L and Novozyme 188 protein concentrations at 50 °C (initial concentrations of 1.61 g Celluclast 1.5 L/L and 1.06 g Novozyme 188/L) is approximately equal to the total protein concentration in the solution of Celluclast 1.5 L and Novozyme 188 at 50 °C (initial concentration of 2.703 g proteins/L) for the period of time monitored. Thus the widely held assumption of constant protein concentration during hydrolysis at 50 °C is invalid due to the apparent precipitation of proteins from Novozyme 188, possibly due to aggregation.

Dekker & Assays (1986) reported that Novozyme 188 lost 17 and 72% of its *β*-glucosidase activity after 48 and 120 h incubation at 50 °C. Chundawat et al. (2011) reported a *β*-glucosidase protein composition of 7.7% in Novozyme 188, with large amounts of amylases (43.6%) and other proteins (42.1%). Therefore, to evaluate if the loss of *β*-glucosidase activity reported by Dekker & Assays (1986) is caused by *β*-glucosidase aggregation and precipitation, the stability of P-*β*-glucosidase from *A. niger* in solution at 50 °C was examined.

### Stability of *β*-glucosidases from *A. niger*

Protein unfolding can be caused by pH changes, organic solvents, heat, protein concentration, shaking, or the presence of other proteins or chemical compounds (Lehninger, Nelson & Cox, 2005). It is hypothesized that native enzyme or protein monomer undergoes a conformational change commonly called denaturation. The protein denaturation can, in some cases, lead to the exposure of “sticky” hydrophobic areas. These areas increase the propensity of the monomer to aggregate or “stick” to each other, causing it to become “active” (Morris, Watzky & Finke, 2009). The active monomers or unfolded proteins begin to aggregate forming oligomers that ultimately lead to insoluble fibrils or amorphous aggregates.

In his study of the thermal denaturation of almond *β*-glucosidase using differential scanning calorimetry (DSC), Tanaka (1991) found evidence indicating that *β*-glucosidase undergoes an irreversible structural transformation from its native conformation to a denatured form between pH 4–8 and 50–90 °C. Therefore, given the changes in Novozyme 188 protein concentration in this study at similar temperatures, the *β*-glucosidases in Novozyme 188 may suffer a similar conformational change leading to aggregation and, subsequently, precipitation. In order to develop a kinetic model of *β*-glucosidase aggregation, we utilized advances made in the field of heat induced *β*-lactoglobulin aggregation and precipitation, a phenomenon well-studied by the food industry (Verheul, Roefs & De Kruif, 1998).

The proposed protein aggregation reaction consists of two steps: the denaturation and the aggregation steps (Verheul, Roefs & De Kruif, 1998). The denaturation step accounts for *β*-glucosidase thermal denaturation, which was assumed to be an irreversible reaction (Tanaka, 1991):

(1) \begin{eqnarray*} B\xrightarrow{{k}_{1}}{D}^{\ast } \end{eqnarray*}

The native *β*-glucosidase (*B*) changes its structure as a result of high temperature, producing a denatured form of the enzyme (*D*^∗^) that then aggregates in a series of irreversible aggregation reactions. It was assumed that only denatured enzymes participate in the aggregation step. These aggregation reactions can be represented as:

(2) \begin{eqnarray*} \begin{array}{@{}l@{}} \displaystyle {D}^{\ast }+{D}^{\ast }\xrightarrow{{k}_{1}}{D}_{n}^{\ast }\\ \displaystyle {D}^{\ast }+{D}_{n}^{\ast }\xrightarrow{{k}_{n}}{D}_{n+1}^{\ast }\\ \displaystyle \vdots \\ \displaystyle {D}_{n}^{\ast }+{D}_{n+1}^{\ast }\xrightarrow{{k}_{m}}{D}_{m}^{\ast } \end{array} \end{eqnarray*}

${D}_{n}^{\ast },{D}_{n+1}^{\ast }$ and ${D}_{m}^{\ast }$ are denatured *β*-glucosidase polymers consisting of *n*, *n* + 1, and *m* monomeric units (*n*, *m* ≥ 2) and are treated as equivalent species. The aggregation step is comprised of hundreds of autocatalytic reactions (Morris, Watzky & Finke, 2009) where denatured polymers adsorb on the aggregate increasing its size and surface area. In consequence, the number of active spots to which denatured polymers can “stick”, increases with the growing aggregate. Therefore, the aggregation of each polymer catalyzes the next aggregation reaction and, consequently, the entire aggregation process. In consequence, the aggregates ${D}_{n}^{\ast },{D}_{n+1}^{\ast }$ rapidly grow to form the insoluble species ${D}_{m}^{\ast }$. According to the Bodenstein principle (Helfferich, 2003), a steady-state situation will be reached quickly once the aggregation reaction starts; the rate at which *D*^∗^ is formed in the denaturation step will equal the rate at which it disappears in the aggregation step (Roefs & De Kruif, 1994). Therefore, the thermal denaturation step (Eq. (1)) is slower than the aggregation step and is thus the rate limiting step. Under this consideration, our analysis assumes only native enzymes to be soluble. From this reaction, assuming first order kinetics, the *β*-glucosidase rate of disappearance is given by:

(3) \begin{eqnarray*} -\frac{d[B]}{d t}={k}_{1}[B] \end{eqnarray*}

Integrating this equation and taking [*B*] = [*B*_0_] (g/L) at *t* = 0 gives:

(4) \begin{eqnarray*} [B]=[B_{0}]{e}^{-{k}_{1}t} \end{eqnarray*}

**Figure 4:**
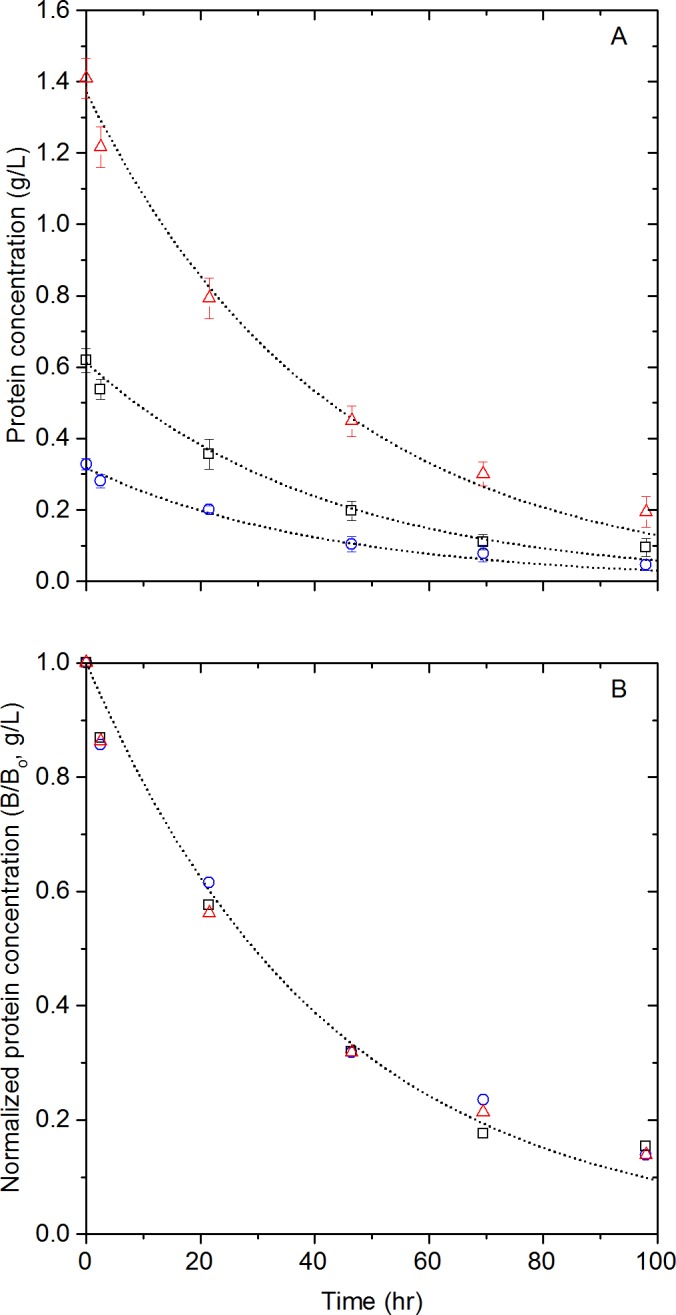
Lyophilized *β*-glucosidase protein concentration changes. Lyophilized *β*-glucosidase concentration (A) and normalized concentration (B) at 50 °C, experimental data ([*B*_0_] = 0.317 g/L (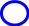), 0.612 g/L (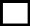) and 1.388 g/L (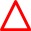)) and 1^st^ order kinetic model, Eq. (4) (
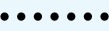
).

To check the validity of the model, solutions of different P-*β*-glucosidase concentrations were prepared using powdered P-*β*-glucosidase and incubated at 50 °C. Soluble protein concentration was monitored and, as shown in Fig. 4, protein concentration decreased by as much as 84% over 98 h. The protein concentrations shown in Fig. 4 were used to fit Eq. (4). The measured initial protein concentrations were used in the model fitting process. The rate constant was determined by least squares regression, obtaining a *k*_1_ of 0.024 h^−1^. The proposed model had a good fit to the aggregation of P-*β*-glucosidase experimental data with a *R*^2^ = 0.97 as shown in Fig. 4A. The analysis of the residuals showed that 78% of the predicted P-*β*-glucosidase concentrations are within one standard deviation of the experimental data. In agreement with the model, the aggregation of P-*β*-glucosidase does not depend on the initial enzyme concentration as shown in Fig. 4B.

Based on the presented model, 68% of the initial concentration of P-*β*-glucosidase is lost after 48 h incubation at 50 °C. However, Dekker & Assays (1986) reported an activity loss of 17% for Novozyme 188 and that enzyme activity is linearly related to enzyme concentration. The reported loss in activity is incongruous with the large loss of P-*β*-glucosidase observed after 48 h incubation; therefore, *β*-glucosidases in Novozyme 188 seems to be more stable than P-*β*-glucosidase. As both *β*-glucosidases come from *A. niger*, the higher apparent stability of *β*-glucosidases in Novozyme 188 may be caused by the stabilizing effect of the other proteins and compounds in Novozyme 188.

### Novozyme 188 stability

**Figure 5:**
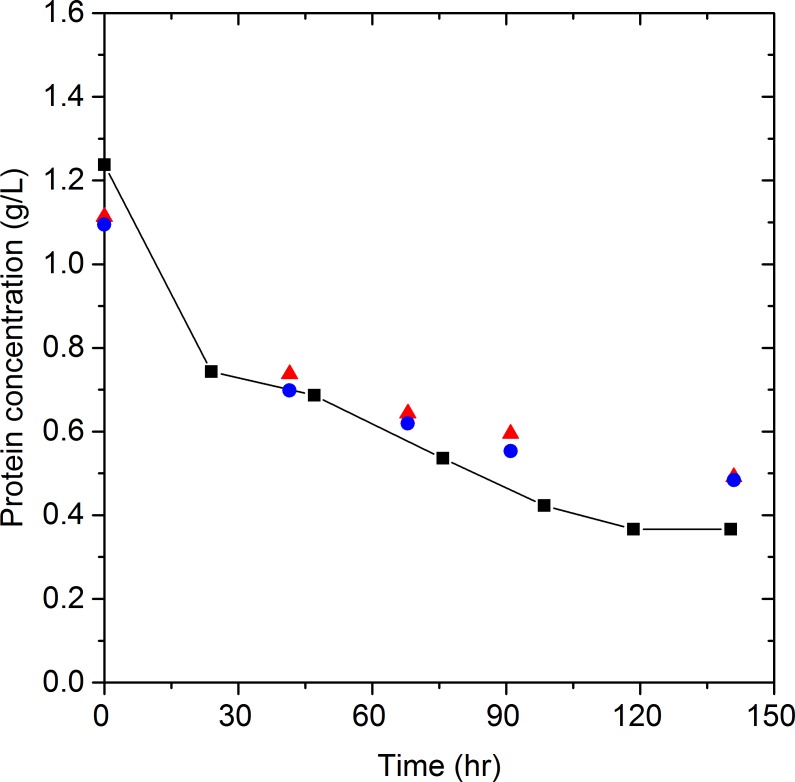
Effect of pretreated biomass on Novozyme 188 protein aggregation. Novozyme 188 protein concentration at 50 °C as a function of time in the absence (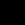) and presence of pretreated biomass: substrate M (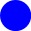) and S (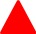). Line is added to assist in visualizing trend.

Understanding Novozyme 188 protein concentration changes under hydrolysis conditions can provide valuable information about the adsorption–desorption process of endo- and exoglucanases. The interaction of Novozyme 188 with pretreated biomass has to be determined, for this reason, Novozyme 188 protein concentration was monitored in the absence and presence of pretreated wheat straw with 4.7% (substrate S) and 9.0% (substrate M) lignin. Figure 5 shows that during the incubation of Novozyme 188 at 50 °C the protein concentration profiles in the absence or presence of pretreated biomass were similar and independent of pretreated biomass lignin composition. These results confirm that, independent of lignin concentration, the proteins and enzymes contained in Novozyme 188 do not adsorb to substrate. The results also confirm that the presence of substrate has no impact on the aggregation and precipitation of Novozyme 188 protein.

The changes in total protein concentration in solution during enzymatic hydrolysis are most probably caused by Novozyme 188 protein aggregation and adsorption–desorption of exo- and endoglucanases in the substrate. The protein mass balance in solution during hydrolysis is:

(5) \begin{eqnarray*} [{T}_{E}]=[{E}_{L}]+[N] \end{eqnarray*}

where the total protein concentration [*T_E_*] (g/L) is defined by the protein concentration of Celluclast 1.5 L in solution [*E_L_*](g/L) and Novozyme 188 protein concentration [*N*] (g/L) as shown in Fig. 3. Hu et al. (2013) reported that the major cellulase monocomponents within Celluclast 1.5 L, on a protein weight basis, were *T. reesei* Cel7A, Cel6A, Cel7B, and Cel5A comprising approximately 56%, 12%, 5%, and 6% of the total protein, respectively. However, more cellulases may be present in the cocktail as the *T. reesei* genome also includes Cel12, Cel61 and Cel45, as reported by Martinez et al. (2008). From these papers, it can be assumed that Celluclast 1.5 L is primarily composed of endo- and exoglucanases. Therefore, the concentration of endo- and exoglucanases in solution [*E_L_*] (g/L), and consequently those adsorbed on substrate, can be indirectly determined by subtracting the Novozyme 188 protein concentration [*N*] (g/L) from the total protein concentration [*T_E_*] (g/L) measured during hydrolysis. This approximation is possible only if the Novozyme 188 protein concentration can be predicted over the course of the enzymatic hydrolysis.

In order to model the aggregation of Novozyme 188, it was assumed that the majority of proteins and enzymes in Novozyme 188 have the same thermal stability. This assumption is supported by the results presented in Fig. 3, where 80% of the initial Novozyme 188 protein concentration is lost after 4 days incubation at 50 °C showing a consistent response to temperature. As mentioned, some compounds present in Novozyme 188 may stabilize the proteins and enzymes at high temperatures. Due to the reported *A. niger* capacity to produce chaperones (Guillemette et al., 2007; Lu et al., 2010), it was assumed that the proteins and enzymes in Novozyme 188 are stabilized by chaperones. Chaperones are proteins that bind to non-native conformations of proteins and assist them in achieving their native structure. Most chaperones bind to exposed hydrophobic surfaces of non-native species and thereby stabilize the protein or enzyme against aggregation. After being released from the chaperone, the protein may proceed to fold correctly, or rebind to a chaperone until a native conformation is reached (Liberek, Lewandowska & Zietkiewicz, 2008; Schlieker, Bukau & Mogk, 2002).

The thermal denaturation of protein in Novozyme 188 can be described following the same assumptions and considerations used for the aggregation of P-*β*-glucosidase. However, a reverse reaction was added to the model to account for the stabilizing effect of chaperones:

(6) \begin{eqnarray*} N{~}_{\longleftarrow _{{k}_{2}}}^{\xrightarrow{{k}_{1}^{{\prime}}}}~{D}^{\ast } \end{eqnarray*}

where *N* refers to the native proteins in Novozyme 188 and D^*^ refers to the denatured proteins which interact with chaperones that regenerate their native protein structure. Therefore, the rate of disappearance and formation of native proteins is:

(7) \begin{eqnarray*} -\frac{d[N]}{d t}={k}_{1}^{{\prime}}[N]-{k}_{2}[{D}^{\ast }] \end{eqnarray*}

The amount of protein precipitated is given by [D^*^] = [*N*_0_]−[*N*] (g/L), where [*N*_0_] is the initial Novozyme 188 protein concentration (g/L). Solving this equation taking [*N*] = [*N*_0_] (g/L), at *t* = 0 yields:

(8) \begin{eqnarray*} [N]=\frac{[{N}_{0}]\left({k}_{2}+{k}_{1}^{{\prime}}{e}^{-t\left({k}_{2}+{k}_{1}^{{\prime}}\right)}\right)}{{k}_{2}+{k}_{1}^{{\prime}}} \end{eqnarray*}

**Figure 6:**
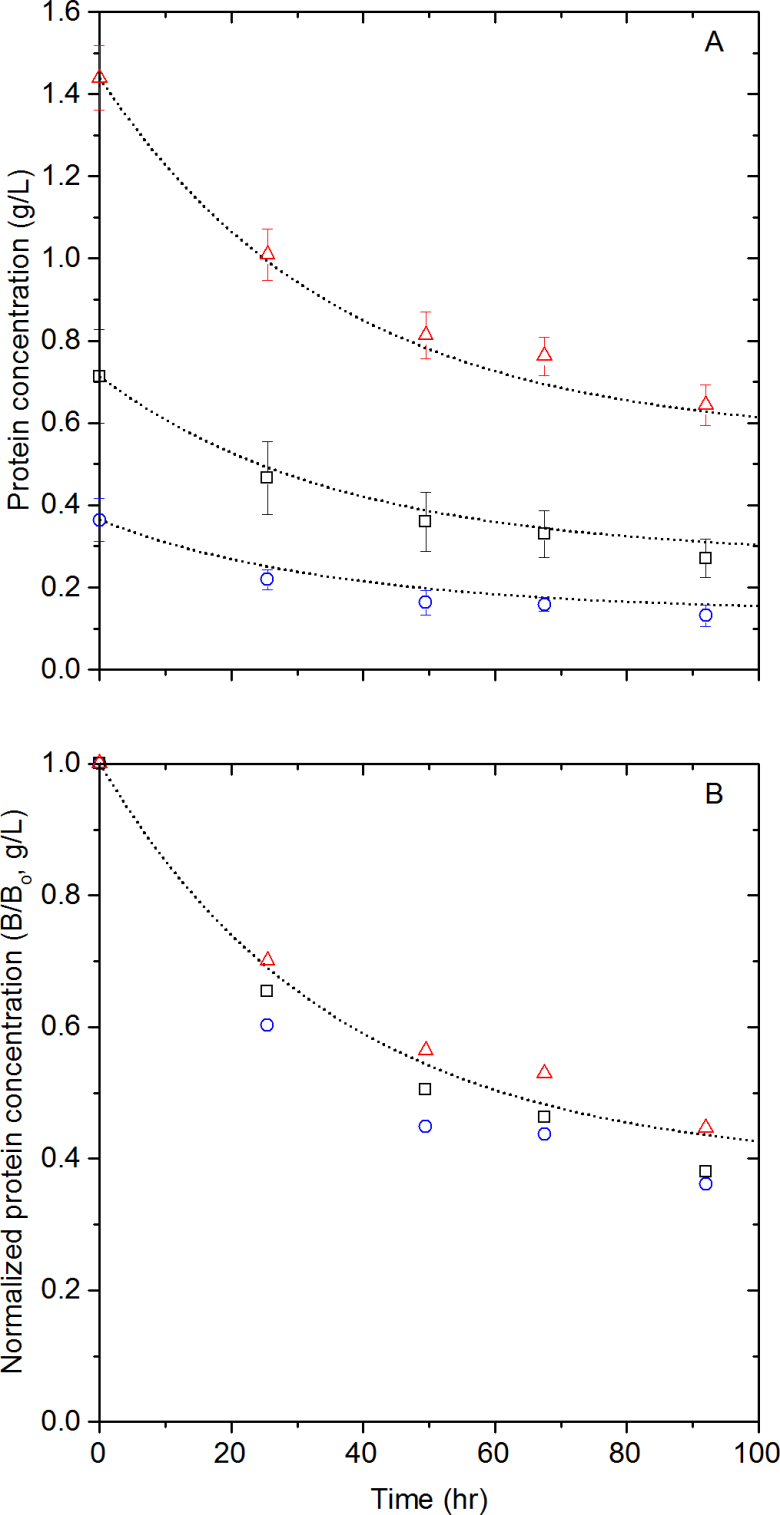
Proposed Novozyme 188 kinetic model incorporating the presence of chaperones. Novozyme 188 concentration (A) and normalized concentration (B) at 50 °C: experimental data ([*N*_0_] = 0.36 g/L (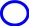), 0.71 g/L (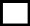) and 1.44 g/L (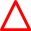)) and the chaperone model, Eq. (8) (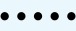).

The rate constants in Eq. (8) were determined by least squares regression to be ${k}_{1}^{{\prime}}=0.017~{\mathrm{h}}^{-1}$ and *k*_2_ = 0.011 h^−1^. The measured initial concentrations were used for the model fitting. Equation (8) successfully predicts the Novozyme 188 protein concentration in solution at the tested hydrolysis conditions with a correlation coefficient of 0.96, as shown in Fig. 6A. Most of the predicted protein concentrations (75%) are within one standard. Therefore, the proposed model (Eq. (8)) has a good fit to the experimental data in the range of concentrations tested.

However, it is clear from Fig. 6B that the protein aggregation from Novozyme 188 exhibits a peculiar behavior which is not captured by Eq. (8): the rate of aggregation was slower at higher initial concentrations. These results contradict the aggregation behavior reported for other systems of proteins (Roefs & De Kruif, 1994; Verheul, Roefs & De Kruif, 1998), where the rate of aggregation and precipitation increases with initial concentration (Wang, 2005). Under severe stress conditions, protein aggregation occurs even in the presence of chaperones because the substantial increase in misfolded proteins cannot be buffered by the limited chaperone capacity (Schlieker, Bukau & Mogk, 2002). Therefore, it is unlikely that the increasing protein stability at increasing Novozyme 188 concentrations is caused by the chaperones.

Other compounds have been explored for their ability to achieve similar stabilizing effects during denaturation. A common method to stabilize proteins is the use of additives such as sugars, polyols, salts, and surfactants, which have chaperone-like effects. These additives stabilize proteins and suppress aggregation by changing the environmental properties of the proteins (Wang, 2005). Novozyme 188 is a mixture of compounds selected to improve enzyme performance and stability. As Novozyme 188 is a commercial product, its preparation and composition are not public knowledge making it difficult to fully understand its behavior. However, the presence of additives in Novozyme 188 may explain the increasing Novozyme 188 stability at high protein concentrations. If additives are present in the cocktail, their concentration increases in proportion to that of Novozyme 188 protein. Therefore, the additives effect in the system becomes stronger as concentration increases.

The hypothetical presence of additives was included in the reverse reaction of the proposed model:

(9) \begin{eqnarray*} N~{\text{}}_{\longleftarrow _{{k}_{2},{A}_{0}}}^{\xrightarrow{{k}_{1}^{{\prime\prime}}}}~{D}^{\ast } \end{eqnarray*}

The denatured proteins (D^*^) are influenced by the additives (*A_o_*) to regenerate their native structure (*N*). Therefore the rate of disappearance and formation of native protein is now:

(10) \begin{eqnarray*} -\frac{d[N]}{d t}={k}_{1}^{{\prime\prime}}\left[N\right]-{k}_{2}[A_{0}][{D}^{\ast }] \end{eqnarray*}

The concentration of additives [*A*_0_] (g/L) was assumed constant and proportional to the initial Novozyme 188 protein concentration [*N*_0_] (g/L), as both come from the same solution. The initial additive concentration was assumed to be given by [*A*_0_] = *m_c_*[*N*_0_], where *m_c_* is a dimensionless constant. Using these relations, Eq. (10) becomes:

(11) \begin{eqnarray*} -\frac{d[N]}{d t}={k}_{1}^{{\prime\prime}}[N]-{k}_{2}{m}_{c}([{N}_{0}]-[N])[{N}_{0}] \end{eqnarray*}

Solving this equation using *k_s_*=*k*_2_*m_c_* and taking [*N*] = [*N*_0_] (g/L) at *t* = 0 yields:

(12) \begin{eqnarray*} [N]=\frac{[N_{0}]\left({k}_{s}[N_{0}]+{k}_{1}^{{\prime\prime}}{e}^{-t\left({k}_{1}^{{\prime\prime}}+{k}_{s}[N_{0}]\right)}\right)}{{k}_{1}^{{\prime\prime}}+{k}_{s}[N_{0}]} \end{eqnarray*}

**Figure 7:**
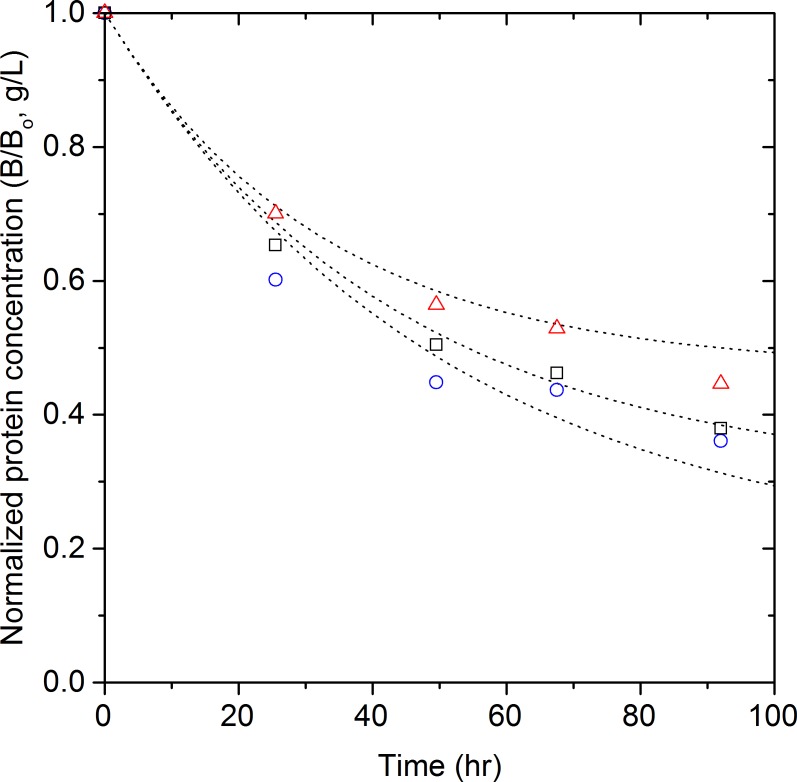
Proposed Novozyme 188 kinetic model incorporating the presence of additives. Normalized Novozyme 188 concentration at 50 °C: experimental data ([*N*_0_] = 0.36 g/L (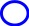), 0.71 g/L (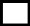) and 1.44 g/L (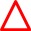)) and the additives model, Eq. (12) (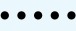).

The rate constants in Eq. (12) were determined by least squares regression to be ${k}_{1}^{{\prime\prime}}=0.016~{\mathrm{h}}^{-1}$ and *k_s_* = 0.008 Lg^−1^ h^−1^. The experimental initial concentrations were used for the model fitting. The value of constant ${k}_{1}^{{\prime\prime}}$ is similar to the value obtained for ${k}_{1}^{{\prime}}$ in Eq. (8), therefore only the reverse reaction is dependent on the presence of additives and thus cocktail loading. Equation (12) successfully predicts the concentration of Novozyme 188 in solution with a correlation coefficient of 0.98, as shown in Fig. 7. The residual analysis showed that all the predicted protein concentrations are now within one standard deviation. Based on the residual analysis for Eqs. (8) and (12), it was concluded that the model with the additives consideration (Eq. (12)) more accurately represents the protein aggregation observed. This conclusion agrees with the second order Akaike’s information criterion (AICc) (Hurvich & Tsai, 2007) calculated for Eq. (8) (AICc = −5.4) and Eq. (12) (AICc = −6.1), where the minimum value reflects a better fit to experimental data.

The predicted aggregation of Novozyme 188 with the additives model presented in Fig. 7 is in better agreement with the experimental results that Novozyme 188 stability increases at high loadings. The model has a good fit to the data at [*N*_0_] = 0.71 and 1.44 g/L, however, the model poorly predicted the protein aggregation at [*N*_0_] = 0.36 g/L, as can be seen in Fig. 7. At low cocktail loadings ([*N*_0_] = 0.36 g/L), the effect of additives is likely minimal. However, the additives concentration, as well as their effect on the system, will increase at higher cocktail loadings ([*N*_0_] = 1.44 g/L) thus the additives’ stabilizing effect can be seen only at high protein loadings. This behavior is similar to the reduction in aggregation of lysozyme when the concentration of additives such as glycine ethylester, 2-methoxyethanol, KCl, and spermine is increased (Kudou et al., 2003; Shiraki et al., 2005). The similarity of our results with previous additive studies supports our hypothesis that the increasing stability of Novozyme 188 protein concentration at high concentrations is due to the effect of additives.

## Conclusions

The successful commercialization of lignocellulosic ethanol rests upon optimization of enzyme cocktails and hydrolysis conditions thus enzyme adsorption to biomass, minimum enzyme loadings and enzyme recycling have been examined in numerous studies. These studies have assumed that the protein concentration at hydrolysis conditions remains constant and that the most significant change in protein concentration during enzymatic hydrolysis is due to adsorption of cellulases on biomass.

In this study, this fundamental assumption was tested by employing two widely used commercial enzyme preparations: Celluclast 1.5 L and Novozyme 188. The constant protein concentration assumption was found to be invalid when a 46% protein loss from a solution of Celluclast 1.5 L and Novozyme 188 was observed after four days incubation at 50 °C. Loss of Novozyme 188 proteins was determined to be the main cause of this decrease as Celluclast 1.5 L protein concentration only slightly decreased (18% after 4 days) at 50 °C. The protein loss in Novozyme 188 is likely due to heat-induced denaturation which promotes protein aggregation and, ultimately, precipitation.

The aggregation of lyophilized *β*-glucosidase in solution was monitored and successfully modeled by assuming the thermal denaturation reaction was the rate limiting step. However, the high precipitation of lyophilized *β*-glucosidase observed does not agree with the reported loss of *β*-glucosidase activity in Novozyme 188. The apparently higher stability of *β*-glucosidase in the cocktail is believed to be caused by the presence of chaperones in the cocktail. The changes in proteins concentration from Novozyme 188 were modeled considering the presence of chaperones in the cocktail. However, the experimental data exhibited increasing protein stability at high protein loadings, which was not predicted by the model. This behavior was proposed to be caused by additives. The presence of additives was included in the Novozyme 188 aggregation model; this produced a model which successfully describes the decrease in aggregation rate with increasing cocktail loading. The observed Novozyme 188 protein concentration changes are in agreement with previous studies of the aggregation of lysozyme in presence of additives.

The thermal instability of Novozyme 188 has not been previously reported and has significant implications for the study of enzymatic hydrolysis. The total protein concentration has been monitored during hydrolysis in past studies to examine the endo- and exoglucanases adsorption–desorption process. However, given the Novozyme 188 protein aggregation observed in this work, the adsorption–desorption information obtained may not be accurate. Therefore, despite the enzyme preparation, protein aggregation must be taken into account when measuring protein concentrations during enzymatic hydrolysis. By combining the measurement of total protein concentration with the model in this paper, endo- and exoglucanases concentration changes can be more accurately determined. Endo- and exoglucanases concentration profiles will lead to greater understanding of the complex adsorption–desorption process during hydrolysis and to optimizing endo- and exoglucanases loading, consequently, decreasing ethanol production costs. Endo- and exoglucanases concentrations profiles during hydrolysis can also help to select the best time to maximize the amount of recyclable enzymes. The proposed model also enables mass balances to determine the amount of endo- and exoglucanases that can be recycled and build a simulation of the ethanol production process with enzyme recycling to evaluate the economics of such technology for ethanol production.
